# Editorial: Deciphering antimicrobial resistance: genetic insights and perspectives

**DOI:** 10.3389/fcimb.2025.1720241

**Published:** 2025-10-22

**Authors:** Massimiliano Lucidi, Mattia Pirolo, Valerio Baldelli, Daniela Visaggio

**Affiliations:** ^1^ Department of Science, Roma Tre University, Rome, Italy; ^2^ Department of Veterinary and Animal Sciences, University of Copenhagen, Frederiksberg C, Denmark; ^3^ Department of Biosciences, University of Milan, Milan, Lombardy, Italy

**Keywords:** antibiotic resistance genes (ARGs), infectious disease epidemiology, mobile genetic elements (MBEs), plasmids, phages, regulatory perspectives, resistance mechanisms, surveillance

Antimicrobial agents have propelled medicine into the era of modern healthcare. However, the efficacy of antimicrobial therapies is progressively weakening as antimicrobial resistance (AMR) escalates, representing a profound threat to global health that transcends human, veterinary, and environmental boundaries. The development of AMR arises from multiple drivers, including *i*) antimicrobial overuse and misuse, *ii*) insufficient diagnostic practices, and *iii*) the intrinsic adaptability of microbial genomes. As AMR continues to spread, our ability to combat common infections and undertake routine medical interventions safely is increasingly compromised (Antimicrobial Resistance Collaborators, 2022). Addressing this challenge requires comprehensive research into the genetic basis of resistance mechanisms, surveillance of microbial communities across multiple reservoirs, and the development of novel strategies to counteract AMR (Jauneikaite et al., 2023).

The Research Topic presented in *Frontiers in Cellular and Infection Microbiology* provides a comprehensive view of the genetic determinants, epidemiology, and functional consequences of AMR across clinical, veterinary, and environmental settings. These studies highlight the need for One Health strategies, integrating surveillance and interventions across humans, animals, and the environment to curb the global spread of AMR.

In veterinary settings, the study of AMR reveals both environmental and human-mediated pathways for the dissemination of resistant organisms. Moreira da Silva et al. demonstrated that small animal veterinary practices in Portugal are contaminated by multidrug-resistant (MDR) microorganisms, including carbapenem- and methicillin-resistant strains, emphasizing the urgent need for robust infection prevention and control measures. Concerning resistant pathogens, Glambek et al. identified a novel *trexAB* operon in *Streptococcus dysgalactiae*, elucidating the genetic basis of low-grade tetracycline resistance and revealing previously unrecognized mechanisms of AMR in both clinical and veterinary settings. The relevance of veterinary reservoirs extends into food production, as illustrated by Habib et al., who reported widespread MDR and toxigenic methicillin-resistant *Staphylococcus aureus* (MRSA) strains in retail meat and produce in the United Arab Emirates, emphasizing the One Health implications of AMR and the potential for community exposure.

Mechanistic insights into novel resistance determinants further broaden our understanding of AMR evolution and its dissemination across microbial species. Lin et al. characterized *aac(6’)-Iaq*, a new aminoglycoside acetyltransferase from *Brucella intermedia*, which confers resistance to multiple aminoglycosides. At the community level, Peng et al. identified silent intestinal colonization by *tet(X4)*-positive, tigecycline-resistant MDR *Escherichia coli* in healthy people, highlighting hidden reservoirs of last-resort antibiotic resistance beyond clinical settings.

Concerning the hospital settings, clinical isolates continue to offer critical insights into AMR dynamics. Liu et al. described the evolution of ceftazidime-avibactam resistance in hypervirulent ST11-K64 *Klebsiella pneumoniae*, driven by the *bla*_KPC-190_ variant that simultaneously confers resistance and partially restores carbapenem susceptibility. Zhang et al. traced the emergence of NDM-1-positive ST592 *K. pneumoniae*, illustrating plasmid-mediated evolution toward carbapenem-resistant hypervirulence. Similarly, Zheng et al. examined the coexistence of *bla*_KPC-2_ and *bla*_VIM-2_ plasmids in intensive care unit (ICU)-derived pandrug-resistant *Pseudomonas aeruginosa* strains, pointing out their stability and minimal fitness cost, which facilitate dissemination within healthcare environments. Expanding the scope to non-tuberculous mycobacteria, Zhao et al. demonstrated that *Mycobacterium seoulense* isolates exhibit variable susceptibility and genomic diversity, reinforcing the importance of targeted susceptibility testing. In the fungal realm, Wei et al. reported that *Candida albicans* can exhibit paradoxical growth under caspofungin exposure, a phenomenon in which fungal cells resume growth at drug concentrations above the minimum inhibitory concentration. This response is driven by activation of stress-response pathways coupled with segmental aneuploidy, which together confer reversible echinocandin resistance, illustrating how dynamically fungi can adapt their genomes under antimicrobial pressure. Intracellular lifestyle is one strategy some microbes use to evade antimicrobials. In this context, Bao et al. offered a lysosome-centered view of *Mycobacterium tuberculosis* pathogenesis, revealing intracellular survival strategies that indirectly influence therapeutic efficacy and potentially contribute to resistance development.

Finally, longitudinal studies, such as that from Zhu et al., can provide an essential perspective to enhance our understanding of AMR evolution and population dynamics. Indeed, this study analysed the *Serratia marcescens* complex in ICU patients over 11 years, identifying inter-ICU transmission and the accumulation of β-lactamase and carbapenemase genes, highlighting the importance of continuous genomic surveillance

**Figure 1:**
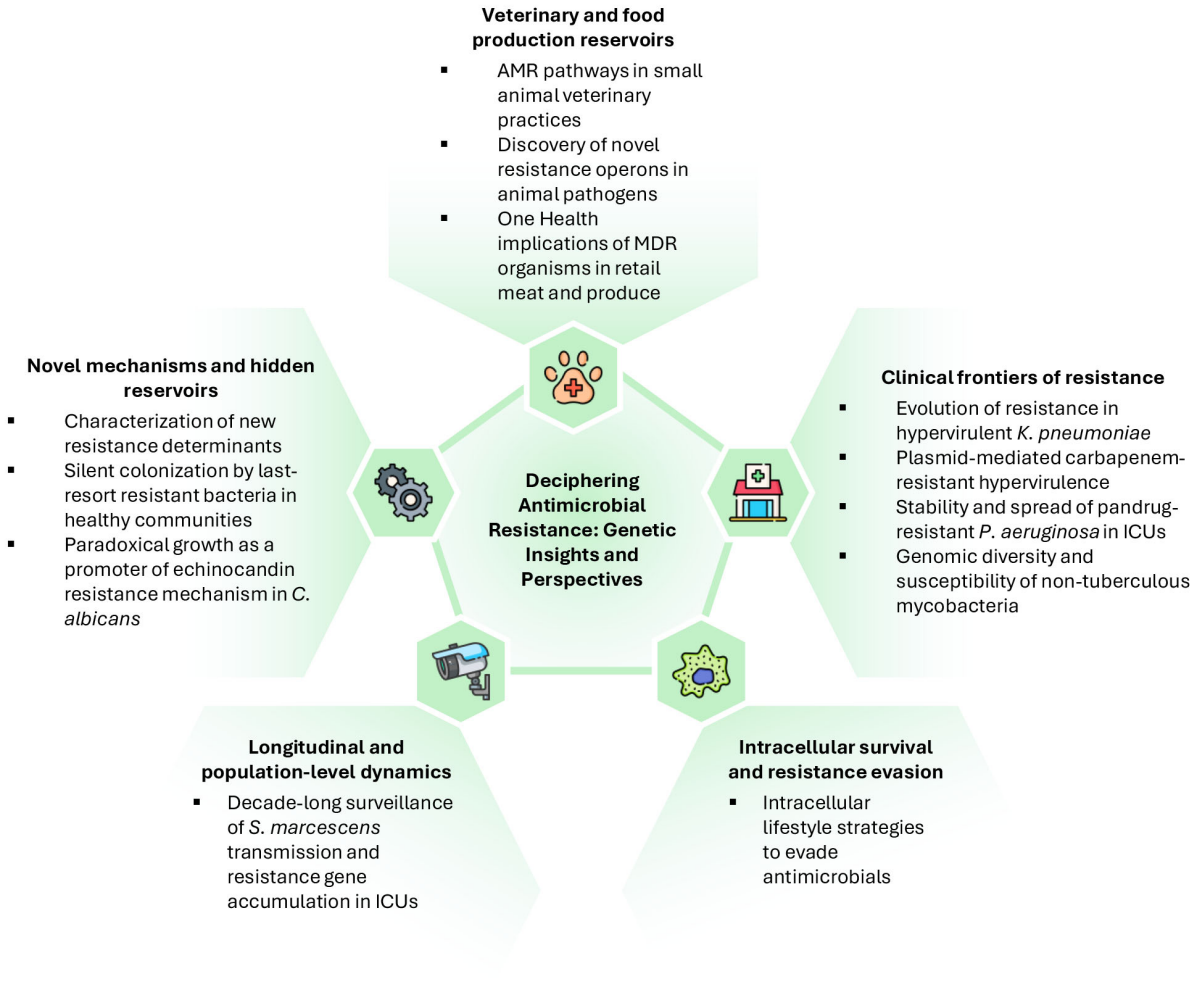
Scientific contributions to this Research Topic.

**Figure 1** highlights the valuable scientific contributions of this Research Topic, exemplifying the breadth and depth of current AMR research. The studies demonstrate that resistance emerges through adaptation to intracellular lifestyle and diverse genetic mechanisms, including plasmid-mediated gene acquisition, point mutations, efflux operons, and genome-scale rearrangements. Surveillance across clinical, community, veterinary, and environmental reservoirs is indispensable to identify emerging threats, while mechanistic studies guide the development of targeted interventions. Additionally, the works highlight the importance of combining genomic, phenotypic, and epidemiological approaches to fully understand the evolution and fitness costs associated with resistance.

Altogether, this Research Topic underscores the urgent need for One Health approaches to combat AMR. It advocates investment in diagnostics, genomic surveillance, and strategies to limit its spread, as deepening our understanding of AMR sharpens surveillance and therapeutic precision while informing policies that bridge human, animal, and environmental health. By fostering interdisciplinary and mechanistic research, the field moves closer to preserving the effectiveness of antimicrobials that underpin modern medicine.
